# Genomic characterization of two carbapenem-resistant *Serratia marcescens* isolates causing bacteremia: Emergence of KPC-2-encoding IncR plasmids

**DOI:** 10.3389/fcimb.2023.1075255

**Published:** 2023-02-08

**Authors:** Junli Jia, Lisha Huang, Long Zhang, Yanbing Sheng, Weili Chu, Hao Xu, Aiguo Xu

**Affiliations:** ^1^ Department of Respiratory, The First Affiliated Hospital of Zhengzhou University, Zhengzhou, China; ^2^ Collaborative Innovation Center for Diagnosis and Treatment of Infectious Diseases, State Key Laboratory for Diagnosis and Treatment of Infectious Diseases, The First Affiliated Hospital, College of Medicine, Zhejiang University, Hangzhou, China

**Keywords:** carbapenem-resistant *S. marcescens*, KPC-2, bacteremia, CTX-M-14, IncR

## Abstract

The occurrence and transmission of carbapenemase-producing-Enterobacterales (CPE) on a global scale has become a major issue. Clinical reports are rarely providing information on the genomic and plasmid features of carbapenem-resistant *Serratia marcescens*. Our objective was to investigate the resistance and transmission dynamics of two carbapenem-resistant *S. marcescens* that are resistant to carbapenem and have caused bacteremia in China. Blood specimens were taken from two individuals with bacteremia. Multiplex PCR was employed to identify genes that code for carbapenemase. Antimicrobial susceptibility tests and plasmid analysis were conducted on *S. marcescens* isolates SM768 and SM4145. The genome of SM768 and SM4145 were completely sequenced using NovaSeq 6000-PE150 and PacBio RS II platforms. Antimicrobial resistance genes (ARGs) were predicted using the ResFinder tool. S1 nuclease pulsed-field gel electrophoresis (S1-PFGE) and southern blotting were employed to analyze plasmids. Two *S. marcescens* that produced KPC-2 were identified from bloodstream infections. The antimicrobial susceptibility testing demonstrated that both of the isolates had a resistance to various antibiotics. The whole-genome sequence (WGS) and plasmid analysis revealed the presence of *bla*_KPC-2_-bearing IncR plasmids and multiple plasmid-borne antimicrobial resistance genes in the isolates. Our comparative plasmid analysis suggested that the two IncR plasmids identified in this study could be derived from a common ancestor. Our findings revealed the emergence of *bla*_KPC-2_-bearing IncR plasmid in China, which could be a hindrance to the transmission of KPC-2-producing *S. marcescens* in clinical settings.

## Introduction

*Serratia marcescens* is a type of Gram-negative bacteria belonging to the order Enterobacterales, family Enterobacteriaceae (Park et al., 2007). *S. marcescens* is frequently encountered in a range of habitats, such as moist areas, prosthetic material, and within the respiratory tract and gastrointestinal flora (Casolari et al., 2013; Rosenthal et al., 2020). Its capacity to cause opportunistic infections in medical facilities is enhanced by these factors. This opportunistic pathogen frequently responsible for hospital-acquired infections, including urinary tract infections (UTIs), respiratory tract infections, conjunctivitis, tear duct infections, and keratitis (Elsherbiny et al., 2018; Konecka et al., 2019; Millan-Lou et al., 2022; Prado et al., 2022; Anderson et al., 2022). *S. marcescens* is a significant hospital-acquired pathogen, and its invasive infections have resulted in high mortality rates (Yeo et al., 2020; do Prado et al., 2021; Bes et al., 2021).

The emergence of acquired antimicrobial resistance (AMR) in *S. marcescens* has become a major health risk to the general public (Prado et al., 2022). There are now several reports of multi-drug resistant (MDR) *S. marcescens* outbreaks carrying either extended-spectrum β-lactamases (ESBLs) or carbapenemases, which confer extended spectrum cephalosporin and carbapenem resistance, respectively (Elsherbiny et al., 2018; Phan et al., 2018; Firmo et al., 2020; Bes et al., 2021; Tamma et al., 2021; Loqman et al., 2021).

In hospital-acquired infections, the prevalence rate of carbapenem-resistant *S. marcescens* has increased recently (Prado et al., 2022). The production of carbapenemase is the primary cause for the rapid and widespread proliferation of *S. marcescens* drug resistance (Firmo et al., 2020; Jimenez et al., 2020). Despite its clinical relevance and the increasing concerns about AMR, little is known about the epidemiology and genetic diversity of carbapenem-resistant *S. marcescens* within healthcare institutions.

Here, we present two cases of bloodstream infections caused by *S. marcescens* isolates that produce KPC-2. We further sequenced two isolates to characterize their genetic diversity, assess for evidence of nosocomial transmission, and determine the genetic context of acquired AMR determinants. These results help the timely implementation of infection control measures.

## Materials and methods

### Bacterial isolation

Identification of the bacterial species was performed by MALDI-TOF MS and 16S rRNA sequence analysis, as described previously (Wang et al., 2019). The isolates were further subjected to PCR to detect the carbapenemase gene as previously described (Zheng et al., 2015).

### Antimicrobial susceptibility testing

Minimum inhibitory concentrations (MICs) of ten antimicrobial agents (imipenem, meropenem, piperacillin-tazobactam, cefotaxime, ceftazidime, aztreonam, ciprofloxacin, gentamicin, amikacin, tobramycin) were determined by agar dilution method, except for colistin and tigecycline, which were determined by the broth microdilution method. Susceptibility was interpreted according to CLSI (2022) and European Committee on Antimicrobial Susceptibility Testing (EUCAST 2022) guidelines (https://www.eucast.org/). *Escherichia coli* ATCC25922 was used as quality control (Wang et al., 2019).

### Whole-genome sequencing and *in silico* analysis

Genomic DNA was extracted using QIAamp DNA Mini Kit (Qiagen, Hilden, Germany) and sequenced using NovaSeq 6000-PE150 (Illumina, San Diego, CA, USA) and PacBio RS II platform (Pacific Biosciences, California, USA). *De novo* assembly was generated by using SPAdes 3.11.0 (Hu et al., 2011). Plasmid replicons and antimicrobial resistance genes were predicted through the PlasmidFinder (https://bitbucket.org/genomicepidemiology/plasmidfinder/src/master/) and ResFinder website (https://cge.cbs.dtu.dk/services/ResFinder/), respectively. The genetic environment surrounding *bla*_KPC-2_ was annotated using RAST3 and Easyfig 2.2.3 (Chi et al., 2019). The complete genome sequences of S. marcescens SM768 and SM4145 were uploaded to NCBI with the following project number: PRJNA841282.

### Plasmid characterization

SM768 and SM4145 were tested by S1 nuclease pulsed-field gel electrophoresis (S1-PFGE) and southern blotting to validate the location of *bla*_KPC-2_. Whole-cell DNA of two isolates was extracted and embedded in gold agarose gel plugs (SeaKem^®^ Gold Agarose, Lonza, Atlanta, GA, USA). The plugs were digested with S1 nuclease (TaKaRa, Dalian, China) and separated by PFGE. Plasmids obtained by PFGE were transferred horizontally to a nylon membrane (Millipore, USA) and hybridized with digoxin-labelled *bla*_KPC-2_-specific probes obtained and the Dig High Prime DNA Labeling and Detection Starter Kit (Roche Diagnostics) (Zheng et al., 2019). The *Salmonella enterica* serotype Braenderup strain H9812 was used as the DNA marker.

### Conjugation assays

Transfer of *bla*_KPC-2_ was investigated using conjugation for isolates SM768 and SM4145. The recipient strains for this experiment were *E. coli* J53 (azide-resistant) and *E. coli* EC600 (rifampicin-resistant), while SM768 and SM4145 were selected as donor strains (Wang et al., 2019).

## Results

### KPC-2-producing *S. marcescens* isolations and patients

Samples of blood from a 36-year-old female and a 49-year-old male yielded two isolates of *S. marcescens*, SM768 and SM4145, both of which were found to be resistant to carbapenems. PCR test for carbapenemase-encoding genes confirmed that both isolates carried the *bla*_KPC-2_ gene.

### Antimicrobial resistance profiles and antimicrobial resistance genes

Isolates SM768 and SM4145 showed resistance to imipenem, piperacillin-tazobactam, cefotaxime, ceftazidime, and aztreonam (**Table S1**). In addition, two isolates were susceptible to amikacin and tobramycin. It is expected that SM768 and SM4145 both exhibited high-level resistance to colistin, as *Serratia* has an intrinsic resistance to this antibiotic. Whole-genome sequencing showed that SM4145 carried multiple antimicrobial resistance genes (ARGs). These include ESBLs resistance gene *bla*_CTX-M-14,_ the plasmid-encoded quinolone resistance gene *qnrS1*, and the aminoglycoside resistance gene *aac6’-Ic*.

### Determination of the *bla*_KPC-2_ gene location and transferability

**Figure 1:**
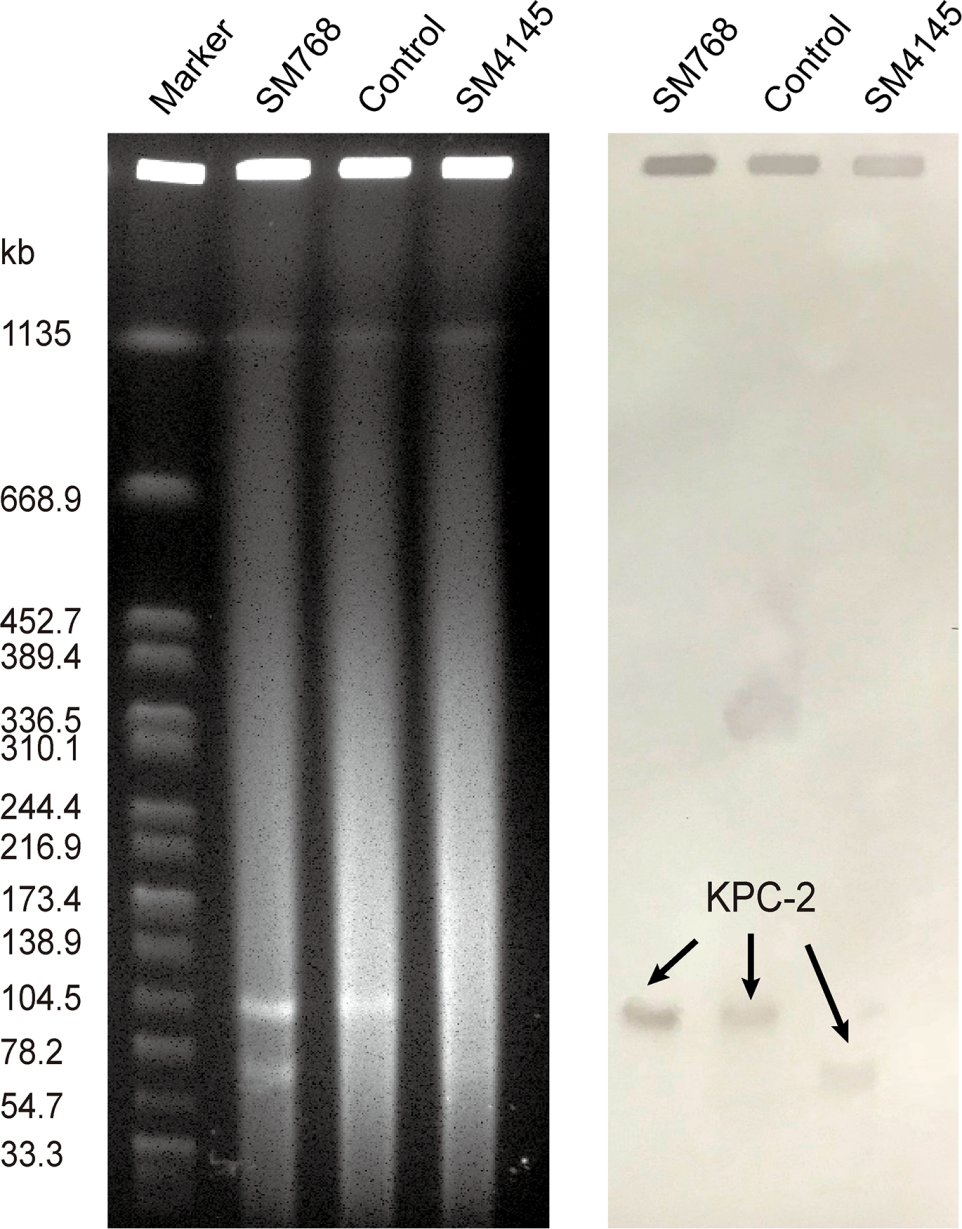
S1-PFGE analysis of KPC-2-producing *S. marcescens* isolates and southern hybridization using a *bla*_KPC-2_ probe. Lane M, molecular weight marker *Salmonella* Braenderup H9812; Lane SM768, isolate *S. marcescens* SM768; Lane control, KPC-2-producing *Klebsiella pneumoniae* isolate 1095 served as the control; Lane SM4145, isolate *S. marcescens* SM4145.

S1-PFGE and Southern blot revealed that SM768 isolate carried a ~105 kb plasmid harbouring *bla*_KPC-2_ gene. In contrast, SM4145 isolate harbouring a ~70 kb plasmid encoding *bla*_KPC-2_ gene (**Figure 1**). Furthermore, the *bla*_KPC-2_ genes could be transferred from SM768 into the recipient *E. coli* strains *via* conjugation, confirmed by PCR. In contrast, SM4145 was negative for the transferability test (data not shown).

### Genetic context of *bla*_KPC-2_ gene

**Figure 2:**
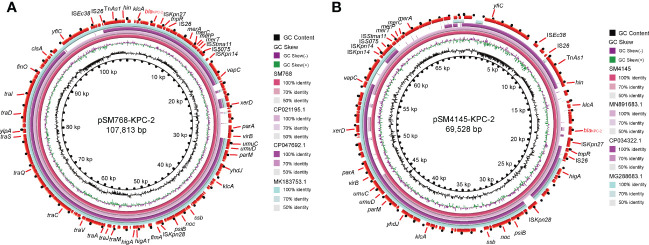
Complete sequences of two *bla*_KPC-2_ *-*harbouring IncR plasmids were recovered in this work. **(A)** Alignment of plasmid sequence of pSM768-KPC-2 with other *bla*_KPC-2_ *-*bearing plasmids pH17-2 (CP021195), pC110-KPC (CP047692), and p17-15-KPC (MK183753). **(B)** Comparison of pSM4145-KPC-2 with plasmids pK033_1 (CP034322), p314013-KPC (MN891683), and pE20-NR (MG288683). Circles inside to outside denote the GC content, GC screw, and the ORFs in both DNA strands. Block arrows represent coding sequences and indicate the direction of transcription. *bla*_KPC-2_ gene is highlighted in red. Arrow size is proportional to gene length. The circular image of multiple plasmids comparisons was generated with the BLAST Ring Image Generator.

The WGS results demonstrated that two *bla*_KPC-2_-encoding plasmids, pSM768-KPC-2, were IncR-type plasmids with 107,813 bp, and pSM4145-KPC-2 was also an IncR-type plasmid with the size of 69,528 bp, respectively (**Figure 2** **)**. Plasmid pSM768-KPC-2 contained a collection of genes involved in segregation, fertility inhibition, stability, and conjugal transfer of the plasmid (*parA*, *parM*, *finO*, *umuCD*, and *tra* regulon), which together constructed the essential backbone of pSM768-KPC-2. In contrast, the *tra* regulon was absent in plasmid pSM4145-KPC-2.

**Figure 3:**
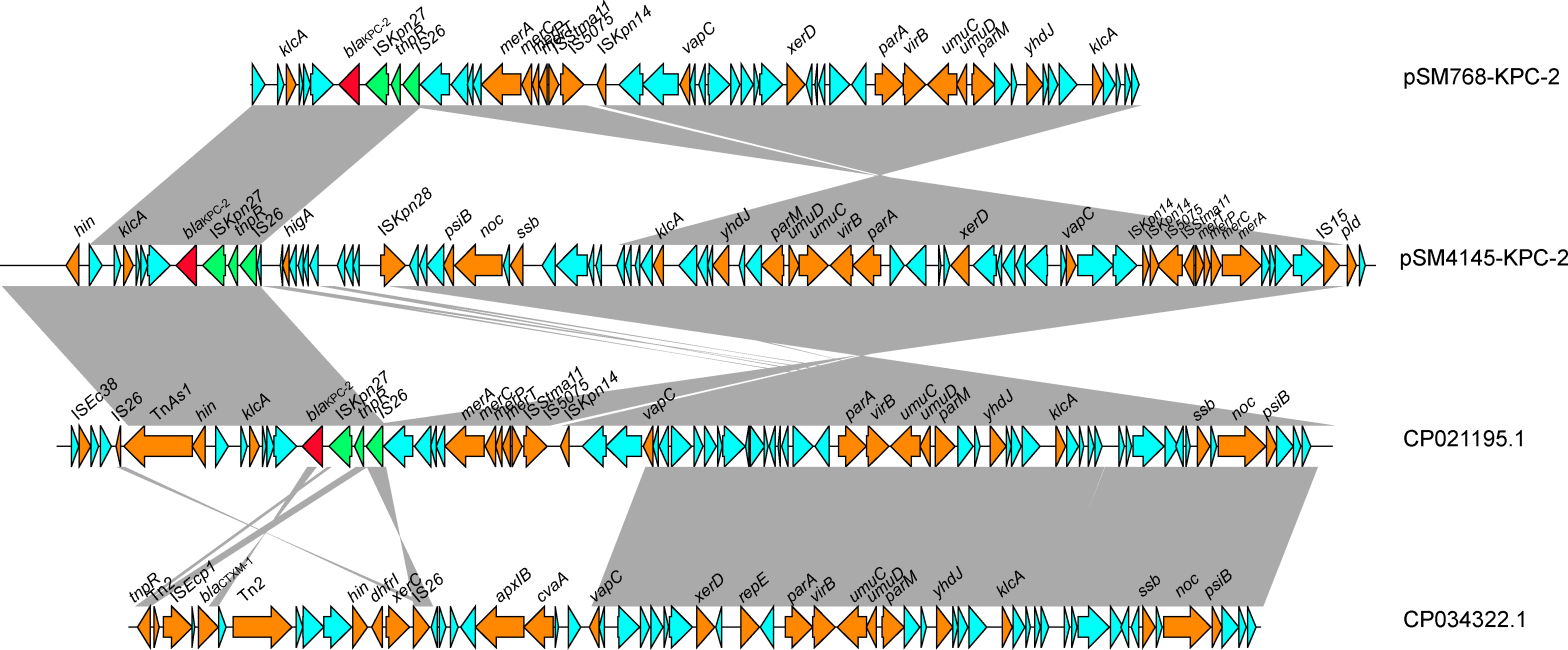
Colinear genome alignment of pSM768-KPC-2, pSM4145-KPC-2, pH17-2, and pK033_1. Arrows represent the direction of transcription. Red open reading frames (ORFs) indicate KPC-2.

The *bla*_KPC-2_ gene surrounding pSM768-KPC-2 and pSM4145-KPC-2 showed the same genetic background. The *bla*_KPC-2_ gene was flanked by an IS*Kpn27*, *tnpR*, and IS*26* elements downstream and a *klcA* gene upstream (**Figure 3**). Comparative plasmid analysis revealed that pSM768-KPC-2 shares a high identity with pSM4145-KPC-2, except a ~38 kb sequence was inserted in pSM768-KPC-2 plasmid. This indicated that pSM768-KPC-2 and pSM4145-KPC-2 might be derived from a common ancestor. In addition, *in silico* analysis also found that pSM768-KPC-2 exhibits high relatedness with a ~107 kb KPC-2-carrying IncR plasmid pH17-2 (CP021195) from *Escherichia coli* and a ~150 kb plasmid pK033_1 (CP034322) from *K. pneunomiae.*

## Discussion

*Serratia marcescens* is a well-known opportunistic pathogen that is often seen to be responsible for infections in intensive care, surgical and dialysis facilities (Ferreira et al., 2020). Antimicrobial resistance of *S. marcescens* has not been widely explored in China (Lin et al., 2016; Su et al., 2017; Huang et al., 2021; Zhong et al., 2022; Hou et al., 2022). Herein, we found two carbapenem-resistant *S. marcescens* from patients with bloodstream infections in China, and the *bla*_KPC-2_ gene, which is native to the area, is likely to be disseminated through the transmission of IncR plasmids.

The global spread of the resistance of Enterobacterales to many antibiotics, seriously affecting the treatment of infections, has become a major public issue (Hu et al., 2018; Dong et al., 2018; Dong et al., 2018; Zheng et al., 2020). The major plasmid types carrying *bla*_KPC-2_ published included IncFII, IncF, IncP-6, IncN, ColRNAI, and IncI2 (Xu et al., 2018; Hu et al., 2019; Xiaoliang et al., 2019; Abril et al., 2021; Peng et al., 2022). To the best of our knowledge, this is the first time that *bla*_KPC-2_ genes have been found to be carried by IncR plasmid in *S. marcescens* isolates from bacteremia.

It is now acknowledged that *S. marcescens* has the potential to cause disease in vulnerable individuals. Although *S. marcescens* displayed relatively low virulence, the emergence of carbapenem-resistant *S. marcescens* has become a real threat to patients (Zhang et al., 2007). In China, the identification of KPC-2-producing *S. marcescens* was first described in 2007 (Zhang et al., 2007). Since then, related reports mainly originated from eastern China (Miao et al., 2018; Li et al., 2018; Xie et al., 2020; Xu et al., 2020), which indicates that regional dissemination of such pathogens might be due to the *bla*_KPC-2_-harbouring plasmids.

The global distribution of CTX-M variants showed that the CTX-M-1 group (especially CTX-M-15) was the dominant genotype in most regions, while the CTX-M-9 group (especially CTX-M-14) has been reported to be the most common genotypes in China in recent years (Zhang et al., 2016; Shen et al., 2017; Huang et al., 2018; Feng et al., 2019; Yuan et al., 2019; Zheng et al., 2021). A previous study found that CTX-M-14 was the most common gene type in *S. marcescens* isolates in China (Yang et al., 2012). Our data is consistent with these findings.

Previous studies have indicated that *S. marcescens* often presents carbapenem resistance caused by plasmids with differing replicon types that contain the *bla*_KPC_ gene (Xu et al., 2020). In this work, we identified and characterized two IncR plasmids from *S. marcescens.* We further employed NovaSeq and PacBio RS II platforms to clarify the genetic context of IncR plasmids, which are acutely lacking for *bla*_KPC_-bearing IncR plasmids. However, the small size of *bla*_KPC_-harbouring IncR plasmids identified in this study is the limitation merit mentioning. A further comprehensive investigation is warranted on the spread of *bla*_KPC_-harbouring IncR plasmid in other Enterobacterales.

To summarize, we characterized KPC-2-producing *S. marcescens* isolates from bloodstream infections in terms of their antimicrobial susceptibility, antimicrobial resistance genes, and plasmid transfer mechanism. Both *bla*_KPC_ genes were located on the IncR plasmid, which presents a potential challenge for interrupting the transmission of KPC-2-producing *S. marcescens* in clinical settings. Our insights may have implications in the clinical care and monitoring of KPC-2-producing bacteria.

## Data availability statement

The datasets presented in this study can be found in online repositories. The names of the repository/repositories and accession number(s) can be found in the article/**Supplementary Material**.

## Author contributions

AX and JJ designed and executed the study, performed the results analyses, and drafted the manuscript. JJ, LH, LZ, and YS handled the molecular experiments. WC and HX established and performed the whole genome sequencing. All authors contributed to the article and approved the submitted version.
