# Supplementary material for: Genomic characterization of two carbapenem-resistant Serratia marcescens isolates causing bacteremia: Emergence of KPC-2-encoding IncR plasmids
Source: Front Cell Infect Microbiol. 2023 Feb 8;13:1075255. doi: 10.3389/fcimb.2023.1075255 (PMC9945258; doi:10.3389/fcimb.2023.1075255)
Supplement: Supplementary file 1 [file Table_1.docx]

**Table S1**. Antimicrobial susceptibility profiles of two KPC-2-producing *Serratia marcescens* isolates.

| **Antibiotics** | **MIC^a^** | |
| --- | --- | --- |
|  | **Isolate SM768** | **Isolate SM4145** |
| Imipenem | 32 | > 64 |
| Meropenem | 2 | 32 |
| Piperacillin-tazobactam | 128 | > 128 |
| Cefotaxime | 32 | 64 |
| Ceftazidime | 8 | 8 |
| Aztreonam | 64 | 64 |
| Ciprofloxacin | 4 | 32 |
| Gentamicin | 64 | 0.5 |
| Amikacin | > 64 | 1 |
| Tigecycline | 2 | 1 |
| Colistin | > 64 | 64 |
| Tobramycin | > 16 | 0.5 |

^a^ MICs were determined according to CLSI (2017) and European Committee on Antimicrobial Susceptibility Testing (EUCAST) (version 8.1, 2019) guidelines.
